# Editorial: Mechanisms of fermented foods and interactions with the gut microbiome

**DOI:** 10.3389/fmicb.2026.1923421

**Published:** 2026-07-20

**Authors:** Arghya Mukherjee, Birsen Yılmaz, Elena Bartkiene, Joao Miguel Rocha

**Affiliations:** 1 Department of Food Biosciences, Teagasc Food Research Centre, Fermoy, Ireland; 2 Tata Institute of Fundamental Research, Hyderabad, India; 3 Department of Food Safety and Quality, Lithuanian University of Health Sciences, Kaunas, Lithuania; 4 Institute of Animal Rearing Technologies, Lithuanian University of Health Sciences, Kaunas, Lithuania; 5 Instituto de Engenharia de Sistemas e Computadores, Tecnologia e Ciência, Porto, Portugal; 6 Laboratório Associado, Escola Superior de Biotecnologia, Centro de Biotecnologia e Química Fina, Universidade Católica Portuguesa, Porto, Portugal

**Keywords:** fermented foods, gut microbiome, gut-organ axes, lactic acid bacteria, probiotics

Fermentation has shaped human diets for millennia, valued first as a means of preserving food and later for enhancing the organoleptic properties of foodstuffs. Only in recent years, however, have we started to understand how fermentation actively reshapes the biological properties of fermented food matrices. Across cultures, fermented foods such as kefir, sauerkraut, and fermented legumes have long been dietary staples, yet how they influence host physiology has not been fully understood. Some of the primary benefits of fermentation come from its transformation of the food matrix to generate new metabolites and, in many cases, from the introduction of beneficial, live microorganisms capable of interacting directly with the gut. This Research Topic, *Mechanisms of fermented foods and interactions with the gut microbiome*, brings together ten contributions—eight research papers and two reviews— that examine these interactions from several angles: how specific fermented foods alter gut microbial composition, how fermentation-derived microbes and metabolites act on distant organs through gut-mediated axes, and how the fermentation process itself determines a food's downstream interaction with the gut. Together, they build a mechanistic picture of why fermented foods matter for gut health, rather than a purely associative one, marking a substantial contribution to a growing body of mechanistic fermented-food research.

Two contributions opened this evidence base with human trials that isolate fermented dairy as the variable of interest. Chonnacháin et al. found that unmelted Cheddar cheese increased fecal bacterial diversity in a controlled 6-week trial, while an equivalent melted-cheese arm did not, with *Dorea, Erysipelotrichaceae* UCG-003, and *Bacteroides* shifting specifically in the unmelted group—evidence that processing state, not fermentation alone, shapes a dairy food's microbial effect. Through a randomized, controlled, parallel-group trial, Choi et al. showed that 2 weeks of kefir consumption (daily 150 mL) in healthy participants aged 18–30 years increased the abundance of lactate-producing taxa, including *Bifidobacterium breve, Ruthenibacterium*

*lactatiformans, Weissella koreensis*, and *Leuconostoc mesenteroides*, along with *Blautia* species, distinguishing kefir's microbial signature from both unfermented milk and yogurt comparators. Together, these studies establish that even within a single food category, fermentation's gut effects depend on how the food is made and consumed.

A second cluster of contributions moves beyond compositional change to trace well-defined mechanistic pathways from fermented-food microbes to organs well beyond the gut. Zhou, Duan et al. isolated three *Lactobacillus* strains (*Lactiplantibacillus* sp. LP03, *Levilactobacillus brevis* LB06, and *Loigolactobacillus coryniformis*) from Chinese sauerkraut juice and tested them in a bleomycin-induced mouse model of pulmonary fibrosis; the most pronounced effects were observed with *Lactiplantibacillus* sp. LP03, which reduced mortality, systemic inflammation, and collagen deposition, restored gut *Akkermansia* and *Ligilactobacillus*, and elevated circulating palmitoylethanolamide—a gut-lung axis originating in a single sauerkraut isolate. Zhou, Zhang et al. reported on the isolation and benefits of the active agent itself: an exopolysaccharide from *Lactiplantibacillus plantarum* NMGL2 that limited body-weight loss, reduced disease activity, and reinforced the colonic mucosa in a dextran sodium sulfate (DSS)-induced colitis mouse model, down-regulating NF-κB signaling and up-regulating the tight-junction proteins ZO-1 and occludin. These findings are significant, as the EPS produced by *Lactiplantibacillus plantarum* NMGL2 alleviated inflammatory bowel disease (IBD) by suppressing the NF-κB signaling pathway, suggesting its potential as a functional food agent for preventing IBD.

At a systems level, the review by Kezer et al. shows how probiotics, prebiotics, and synbiotics act on the gut-brain axis across models of autism, depression, and neurodegeneration, in addition to their potential benefits for immune homoeostasis and the management of chronic diseases, including IBD. Across all three, the through-line is the same: a defined microbe or metabolite, traceable to a fermented food, acting on a specific organ system via the gut.

A third pair of contributions examined fermented foods through a gut-liver lens, using high-fat diet mouse models to connect microbial shifts to systemic lipid handling. Hao et al. found that lactic acid bacteria-fermented triple-bean soup, a traditional Chinese functional food, outperformed its unfermented counterpart in correcting high-fat-diet-induced dysbiosis and dyslipidaemia: it increased short-chain fatty acid-producing genera such as *Prevotella, Coprococcus*, and *Oscillospira*, and raised butyrate and propionate roughly 1.8-fold. Metabolomics analysis further revealed the reprogramming of bile acid and lipid metabolic pathways, manifesting reduced hepatic steatosis and improved blood lipid levels. These findings position this traditional fermented food, studied with modern multi-omics tools, as a promising candidate for managing metabolic liver disease. Xu et al. combined red yeast rice with phytosterol and lycopene and found that the combination lowered LDL cholesterol more effectively than simvastatin in hypercholesterolaemic mice, although the benefit was dose-dependent and warrants further study, while also reducing body weight, an effect the statin did not produce; the mechanism traced back to a shift toward *Bifidobacterium* and away from *Clostridium* and *Ruminococcus*, altering the bile-acid pool in a way that activated the hepatic FXR pathway and increased fecal cholesterol excretion. In both cases, fermentation-associated microbial change precedes and appears to drive the metabolic outcome, rather than merely accompanying it.

Three further contributions shift the focus from finished fermented foods to the mechanism of fermentation itself—why processing a food changes its relationship with the gut before it is even consumed. The review by Panapparambil Sooraj et al. argues that plant proteins, with their lower digestibility and anti-nutritional factors, leave more undigested protein to reach the colon, where bacterial putrefaction generates ammonia, phenols, and other by-products linked to inflammation and gut barrier dysfunction; fermentation degrades those anti-nutritional factors and increases free amino acids, reducing the undigested protein load before it can be putrefied. Therefore, fermentation could be considered a potential way to improve the digestibility of plant proteins and reduce the risk of putrefaction. Yang et al. showed a parallel effect on a plant substrate's chemistry rather than its protein content: fermenting honeysuckle liquid with *Lactobacillus acidophilus* raised total phenols (by 26.48%), flavonoids (22.59%), and chlorogenic acid (33.57%) well above unfermented levels, along with antioxidant and alpha-glucosidase-inhibitory activity. This study showed that *Lactobacillus acidophilus* fermentation could be a potential approach for developing honeysuckle functional products. Rounding out this group, Aziz et al. provided the first genomic characterization of a sauerkraut-derived *Lactiplantibacillus plantarum* strain, HMX2, mapping bacteriocin genes and stress-survival genes relevant to its candidacy as a functional probiotic—foundational work for the kind of strain-level claims made elsewhere in this Research Topic.

Taken together, the ten contributions to this Research Topic contribute to the field's continuing, gradual shift from association toward mechanism. Two studies show that even within a single fermented food, its form and how it is consumed can determine its effect on the gut. Three further studies trace specific microbes or metabolites along defined axes connecting the gut to the lung, the colon's own barrier, and the brain. Two more studies show that fermentation-derived microbial and bile-acid shifts can outperform a standard pharmaceutical on some metabolic endpoints, and two further studies, together with a foundational genomic characterization, explain why fermentation changes a food's gut-interaction potential before it is even eaten. What remains is the harder translational step: converting these strain- and metabolite-level mechanisms into dietary guidance and functional-food design. This will require more controlled human trials of the kind reported here, rather than relying solely on further animal-model evidence. We hope this Research Topic offers a foundation for that next phase of fermented-food research, an area now entering an exciting period of discovery and innovation.

